# Polynucleotides Attenuate Atopic Dermatitis-like Inflammatory Signaling in Keratinocytes and Macrophages

**DOI:** 10.3390/biomedicines14081826

**Published:** 2026-08-13

**Authors:** Ye Jin Ha, Ka Hee Tak, Jong Lyul Lee, Chan Wook Kim, Ik Jun Moon, Yong Sik Yoon

**Affiliations:** 1 Asan Institute for Life Sciences, Asan Medical Center, University of Ulsan College of Medicine, Seoul 05505, Republic of Korea; hayejin@amc.seoul.kr (Y.J.H.); kahee@amc.seoul.kr (K.H.T.); iamleejong@amc.seoul.kr (J.L.L.); crscwkim@amc.seoul.kr (C.W.K.); 2 Division of Colon and Rectal Surgery, Department of Surgery, Asan Medical Center, University of Ulsan College of Medicine, Seoul 05505, Republic of Korea; 3 Department of Dermatology, Asan Medical Center, University of Ulsan College of Medicine, Seoul 05505, Republic of Korea; ikjun.moon@gmail.com; 4 Hae Dermatology Clinic, Seoul 06522, Republic of Korea

**Keywords:** polynucleotides, HaCaT, RAW 264.7, TNF-α/IFN-γ, LPS, inflammatory skin disorder

## Abstract

**Background**: Atopic dermatitis (AD) is a persistent and recurring skin disease characterized by epidermal barrier dysfunction, immune dysregulation, and elevated expression of proinflammatory mediators. We investigated the anti-inflammatory potential of polynucleotides (PN), highly purified DNA biopolymers isolated from salmonid gonads, in keratinocyte and macrophage activation models. **Methods**: RAW 264.7 macrophages were stimulated with lipopolysaccharide (LPS), whereas HaCaT keratinocytes were stimulated with tumor necrosis factor-α (TNF-α) and interferon-γ (IFN-γ). The effects of PN treatment on the production or expression of inflammatory mediators, cytokines, and chemokines were evaluated. Changes in the phosphorylation of mitogen-activated protein kinases (MAPKs) and Janus kinase 1/signal transducer and activator of transcription 3 (JAK1/STAT3) and in the nuclear localization of nuclear factor-κB (NF-κB) were also assessed. **Results**: In LPS-activated RAW 264.7 macrophages, PN treatment significantly suppressed nitric oxide production and downregulated the expression of inducible nitric oxide synthase (iNOS), TNF-α, IL-1β, and IL-8, accompanied by reduced NF-κB nuclear translocation. In TNF-α/IFN-γ-stimulated HaCaT keratinocytes, PN treatment markedly decreased the secretion levels of IL-6, IL-1β, and thymic stromal lymphopoietin. Moreover, PN treatment markedly reduced T-cell-recruiting chemokines, including MDC/CCL22, TARC/CCL17, RANTES/CCL5, and IL-8. Signaling analyses demonstrated that PN treatment attenuated the phosphorylation of key MAPKs (ERK, JNK, and p38) and the JAK1/STAT3 axis. Furthermore, PN treatment markedly reduced NF-κB nuclear translocation. **Conclusions**: These in vitro findings indicate that the anti-inflammatory effects of PN are associated with reduced activation of multiple core signaling pathways governing cytokine and chemokine responses, supporting further investigation of PN in AD and other chronic inflammatory skin diseases.

## 1. Introduction

Atopic dermatitis (AD) is a chronic, relapsing inflammatory skin disease characterized by epidermal barrier disruption and dysregulated immune responses. Impaired barrier integrity increases transepidermal water loss, resulting in dry skin and pruritus. These symptoms trigger persistent scratching, which exacerbates epidermal damage and perpetuates inflammatory cascades through a pathogenic cycle of immune activation and barrier breakdown [1,2,3]. AD is a multifactorial disease influenced by a complex interplay among genetic predisposition, immune imbalances, and environmental triggers, all of which contribute to epidermal barrier dysfunction and aberrant immune responses. These multifactorial interactions play a crucial role in the pathogenesis of inflammatory skin diseases, including AD [1,4].

The immune response in AD involves a broad array of mediators, including nitric oxide (NO), cytokines, and chemokines, which orchestrate both innate and adaptive immune responses. These mediators modulate physiological and pathological processes driven by immune activation and inflammation. Macrophages are key contributors to this milieu, releasing proinflammatory factors such as tumor necrosis factor-α (TNF-α), interleukin-1β (IL-1β), IL-8, and NO [5,6]. In macrophages, lipopolysaccharide (LPS) binding to Toll-like receptor 4 (TLR4) activates nuclear factor-κB (NF-κB) and mitogen-activated protein kinases (MAPKs), including ERK, JNK, and p38, which subsequently drive inducible nitric oxide synthase (iNOS) expression and proinflammatory cytokine production. Upon activation, NF-κB translocates to the nucleus, where its phosphorylated p65 subunit initiates the transcription of proinflammatory genes [7,8]. This signaling axis is therapeutically targetable, as illustrated by studies of extracutaneous inflammation, in which pharmacological reprogramming of macrophage polarization via NF-κB and transforming growth factor-β (TGF-β) signaling resolved tissue injury [9].

Keratinocytes actively participate in the cutaneous inflammatory network of AD. When exposed to TNF-α and interferon-γ (IFN-γ), keratinocytes upregulate the secretion of proinflammatory cytokines and chemokines, thereby reinforcing the immune response [5,10,11,12]. When keratinocytes mount an exaggerated inflammatory response to external stimuli, chronic inflammation develops, leading to the development of AD, which is characterized by intense pruritus [13].

Polynucleotides (PN), highly purified DNA biopolymers extracted from trout gonads [14], are known for their marked hydrophilicity and water-retention capacity. These molecules form a gel matrix that facilitates hydration and tissue repair. PN have been widely applied in regenerative medicine, particularly as dermal fillers and in intra-articular injections. Prior studies indicate that PN promote cell proliferation, migration, and extracellular matrix remodeling, thereby restoring epidermal integrity [15]. Additionally, PN exhibit anti-inflammatory activity in various models of inflammation, including ischemia–reperfusion injury and colitis, potentially via modulation of the Janus kinase/signal transducer and activator of transcription (JAK/STAT) and NF-κB pathways [16,17,18,19]. Achieving sustained control of AD requires interrupting the self-amplifying circuits that drive relapse, rather than merely suppressing effector cytokines transiently. Consistent with this concept, reversal of branched-chain amino acid-driven inflammatory senescence in the skin prevents AD recurrence in preclinical models [20]. We previously demonstrated that PN significantly attenuate AD pathology in a 2,4-dinitrochlorobenzene (DNCB)-induced murine model [21]. Although our previous study demonstrated that topical PN treatment ameliorated clinical and histopathological features in a DNCB-induced mouse model of AD—including skin barrier dysfunction, epidermal hyperplasia, inflammatory cell infiltration, and proinflammatory cytokine expression—it remained unclear whether this anti-inflammatory response results from a direct effect of PN on keratinocytes and macrophages or is an indirect consequence of improved hydration and barrier restoration within the tissue microenvironment. Therefore, we aimed to characterize the direct cellular response to PN in TNF-α/IFN-γ-stimulated HaCaT keratinocytes and LPS-stimulated RAW 264.7 macrophages, and to examine the associated changes in AD-related cytokines, chemokines, and intracellular signaling pathways. Rather than merely reproducing the previous in vivo findings in vitro, we provide a cell-type-resolved molecular extension that may help explain, at least in part, the anti-inflammatory mechanisms of PN observed in vivo. We hypothesized that PN treatment attenuates inflammation by suppressing activation of the NF-κB, MAPK, and JAK/STAT signaling pathways, which are key cascades in the pathogenesis of AD.

## 2. Materials and Methods

### 2.1. Cells and Reagents

The HaCaT human keratinocyte and RAW 264.7 murine macrophage cell lines were obtained from the American Type Culture Collection (ATCC; Manassas, VA, USA) and cultured at 37 °C/5% CO_2_ in Dulbecco’s Modified Eagle’s Medium (DMEM; Gibco™, Thermo Fisher Scientific, Waltham, MA, USA), supplemented with 10% heat-inactivated fetal bovine serum (Gibco™), streptomycin (100 μg/mL), and penicillin (100 U/mL). PN were supplied by BRPHARM (Wonju, Republic of Korea) as an aqueous solution containing 20 mg/mL (2% *w*/*v*) polynucleotide sodium purified from trout gonadal tissues raised in controlled aquaculture facilities. The PN preparation comprises a DNA-derived biopolymer composed of deoxyribonucleotides linked via phosphodiester bonds. The preparation exhibited a purity ≥ 99% and a mean molecular weight ≥ 1000 kDa. Batch-to-batch consistency was maintained according to the manufacturer’s standardized specifications for identity, concentration, purity, pH, osmolality, and molecular weight distribution.

### 2.2. Cytotoxicity Assay

Cell viability was assessed using the Cell Counting Kit-8 (CCK-8, Dojindo Laboratories, Kumamoto, Japan). Briefly, HaCaT and RAW 264.7 cells were seeded in 96-well plates at a density of 1 × 10^4^ cells/well and incubated overnight. Subsequently, the cells were treated with increasing concentrations of PN (0, 100, 200, 500, and 1000 μg/mL) for 24 h. Absorbance was measured at 450 nm using a microplate reader (Tecan, Melbourne, Australia). The percentage of cell viability was calculated as follows: Cell viability (%) = [(mean absorbance in test wells − mean absorbance in blank wells)/(mean absorbance in control wells − mean absorbance in blank wells)] × 100. Although PN at 1000 μg/mL did not significantly reduce cell viability, a modest downward trend in absorbance was observed. Therefore, concentrations of 100, 200, and 500 μg/mL were selected for subsequent functional experiments.

### 2.3. Measurement of Nitric Oxide (NO) Production

RAW 264.7 cells were seeded at a density of 3 × 10^5^ cells/well in 6-well plates and incubated overnight. The cells were pretreated with PN (0, 100, 200, or 500 μg/mL) for 1 h, and then stimulated with 1 μg/mL LPS (Sigma-Aldrich, St. Louis, MO, USA) and incubated for an additional 24 h at 37 °C. Nitrite accumulation in the culture supernatants was quantified as an indicator of NO production using the Griess Reagent System (Thermo Fisher Scientific, Waltham, MA, USA) in accordance with the manufacturer’s instructions.

### 2.4. Enzyme-Linked Immunosorbent Assay (ELISA)

HaCaT cells were seeded at 3 × 10^5^ cells/well in 6-well plates and incubated overnight. The cells were pretreated with PN (0, 100, 200, or 500 μg/mL) for 1 h, followed by stimulation with 10 ng/mL TNF-α/IFN-γ (TI; R&D Systems, Minneapolis, MN, USA) for 24 h. Culture supernatants were collected and analyzed for IL-6, IL-1β, thymic stromal lymphopoietin (TSLP), macrophage-derived chemokine (MDC/CCL22), thymus and activation-regulated chemokine (TARC/CCL17), RANTES (CCL5), and IL-8 via ELISA (MyBioSource, San Diego, CA, USA), according to the manufacturer’s protocols.

### 2.5. Real-Time Reverse Transcription Polymerase Chain Reaction (RT-PCR)

HaCaT and RAW 264.7 cells were seeded in 6-well plates at 3 × 10^5^ cells/well and incubated overnight. After pretreatment with PN (0, 100, 200, or 500 μg/mL) for 1 h, HaCaT cells were stimulated with 10 ng/mL TI, whereas RAW 264.7 cells were stimulated with 1 μg/mL LPS for 24 h. Total RNA was extracted and reverse-transcribed using random primers and SuperScript II Reverse Transcriptase (Thermo Fisher Scientific, Waltham, MA, USA). Real-time RT-PCR was conducted using SYBR Green I Master Mix (Roche, Mannheim, Germany) on a Roche LightCycler 96 system. Glyceraldehyde 3-phosphate dehydrogenase (GAPDH) served as the internal normalization control. Primer sequences are listed in Supplementary Table S1.

### 2.6. Western Blotting

HaCaT and RAW 264.7 cells were seeded in 60 mm dishes at a density of 1 × 10^6^ cells and incubated overnight. Cells were pretreated with PN at concentrations of 0, 100, 200, or 500 μg/mL for 1 h. HaCaT cells were stimulated with 10 ng/mL TI, whereas RAW 264.7 cells were stimulated with 1 μg/mL LPS for the indicated times. Nuclear and cytosolic proteins were extracted after 10 min of stimulation, whereas iNOS expression was assessed after 24 h. All other signaling targets were analyzed after 30 min of stimulation. Cells were lysed using radioimmunoprecipitation assay (RIPA) lysis buffer (Biosesang, Seongnam, Republic of Korea) or NE-PER Nuclear and Cytoplasmic Extraction Reagents (Thermo Fisher Scientific, Waltham, MA, USA). Lysates were clarified by centrifugation at 13,000 × g for 15 min at 4 °C. Protein concentrations were measured using a Bradford protein assay kit (Thermo Fisher Scientific, Waltham, MA, USA). Equal amounts of total protein (10–50 μg) were resolved by sodium dodecyl sulfate–polyacrylamide gel electrophoresis (SDS-PAGE) and transferred onto polyvinylidene difluoride (PVDF) membranes (Millipore, Bedford, MA, USA). Membranes were blocked with 5% skimmed milk in Tris-buffered saline containing 0.1% Tween-20 (TBST) and incubated overnight at 4 °C with the primary antibodies. After washing, horseradish peroxidase (HRP)-conjugated secondary antibodies (goat anti-rabbit IgG, A120-101P; goat anti-mouse IgG, A90-116P; 1:5000; Bethyl Laboratories, Montgomery, TX, USA) were added, and signals were visualized using ECL substrate (Thermo Fisher Scientific, Waltham, MA, USA). Band intensities were analyzed using ImageJ (version 1.54g, National Institutes of Health, Bethesda, MD, USA). The primary antibodies used were as follows: ERK1/2 (#9102, 1:5000), phospho (p)-ERK1/2 (#9101, 1:5000), JNK (#9252, 1:5000), p-JNK (#4688, 1:2000), p38 (#9212, 1:2000), p-p38 (#4511, 1:2000), JAK1 (#3344, 1:2000), p-JAK1 (#3331, 1:5000), and p-STAT3 (#9145, 1:5000) (all from Cell Signaling Technology, Danvers, MA, USA); STAT3 (sc-8019, 1:2000), NF-κB (sc-8008, 1:5000), and Lamin B1 (sc-374015, 1:1000) (all from Santa Cruz Biotechnology, Dallas, TX, USA); iNOS (PA1-036, 1:2000, Thermo Fisher Scientific, Waltham, MA, USA); and β-actin (A300-4941, 1:5000, Bethyl Laboratories, Montgomery, TX, USA).

### 2.7. Immunofluorescence Assay

HaCaT and RAW 264.7 cells were seeded in a Nunc™ Lab-Tek™ Chamber Slide (Thermo Fisher Scientific, Waltham, MA, USA) at 2 × 10^4^ cells/well and pretreated with PN (0, 100, 200, or 500 μg/mL) for 1 h. HaCaT cells were then stimulated with 10 ng/mL TI, whereas RAW 264.7 cells were stimulated with 1 μg/mL LPS. After 10 min, the cells were fixed with 2% paraformaldehyde in phosphate-buffered saline (PBS) for 15 min at room temperature and washed three times with cold PBS. The fixed cells were permeabilized with 0.2% Triton X-100 in PBS and blocked with 1% bovine serum albumin (BSA) in PBS. After incubation with primary NF-κB antibody (sc-8008, 1:100), cells were incubated with an Alexa Fluor 488-conjugated secondary antibody (RSA1141, 1:500; BioActs, Incheon, Republic of Korea). Nuclei were counterstained with 4′,6-diamidino-2-phenylindole (DAPI, Sigma-Aldrich, St. Louis, MO, USA), and fluorescence images were captured using an Olympus IX71 fluorescence microscope (Olympus Optical Co., Tokyo, Japan).

### 2.8. Statistical Analysis

All experiments were performed independently in triplicate. Data are expressed as the mean ± standard deviation (SD). Statistical significance was evaluated using one-way analysis of variance (ANOVA), followed by Tukey’s post hoc test for multiple comparisons among the experimental groups. All statistical analyses were performed using SPSS v21 (IBM Corp., Armonk, NY, USA). A *p*-value < 0.05 was considered statistically significant.

## 3. Results

### 3.1. Cytotoxic Effects of PN in HaCaT and RAW 264.7 Cells

**Figure 1:**
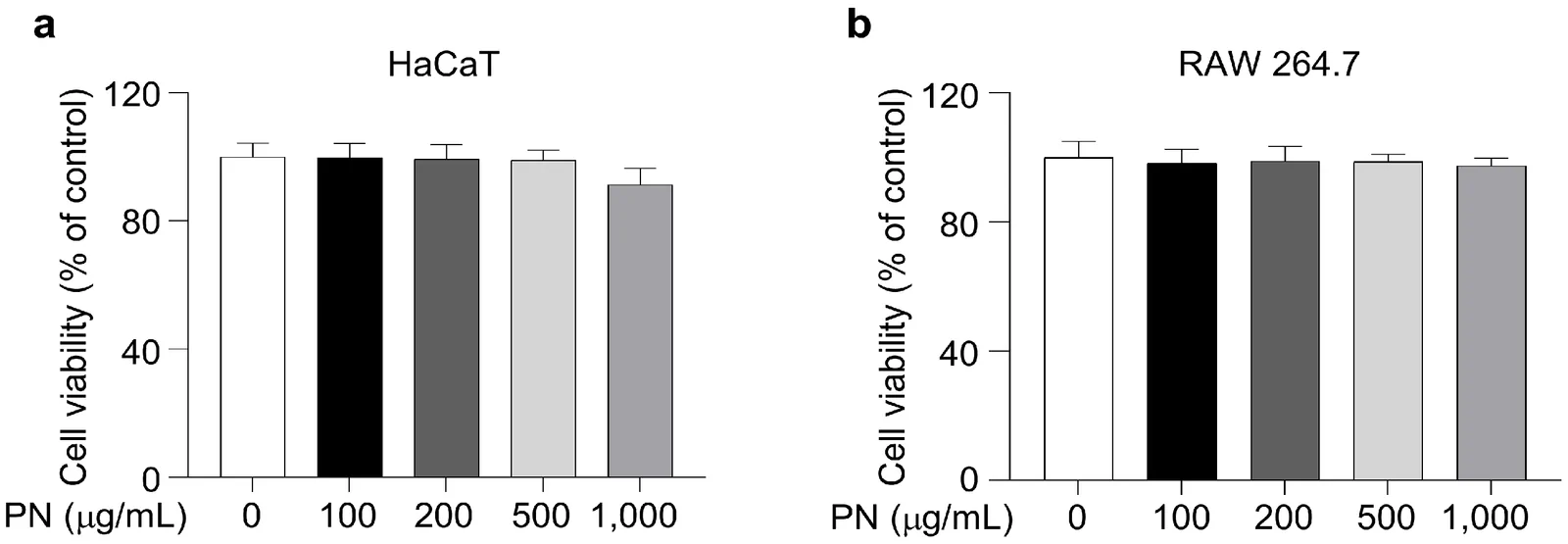
Cytotoxic evaluation of polynucleotides (PN) in HaCaT and RAW 264.7 cells. (**a**) HaCaT and (**b**) RAW 264.7 cells were seeded at 1.0 × 10^4^ cells/well in 96-well plates and incubated overnight. The cells were then treated with the indicated concentrations of PN (0, 100, 200, 500, and 1000 μg/mL) for 24 h. Cell viability was determined using a Cell Counting Kit-8 (CCK-8) assay. Data are presented as the mean ± standard deviation (SD) of three independent experiments.

To assess the cytotoxicity of PN, HaCaT and RAW 264.7 cells were treated independently with PN (0, 100, 200, 500, or 1000 μg/mL) for 24 h. PN treatment did not significantly affect the viability of either cell line compared to that of the untreated group (Figure 1a,b). At 1000 μg/mL, cell viability remained at 90% of the untreated control in HaCaT cells; this reduction was not statistically significant, despite a slight downward trend. Based on these findings, PN concentrations of 100–500 μg/mL were used for subsequent experiments.

### 3.2. Inhibitory Effects of PN on Inflammatory Mediators in LPS-Stimulated RAW 264.7 Cells

**Figure 2:**
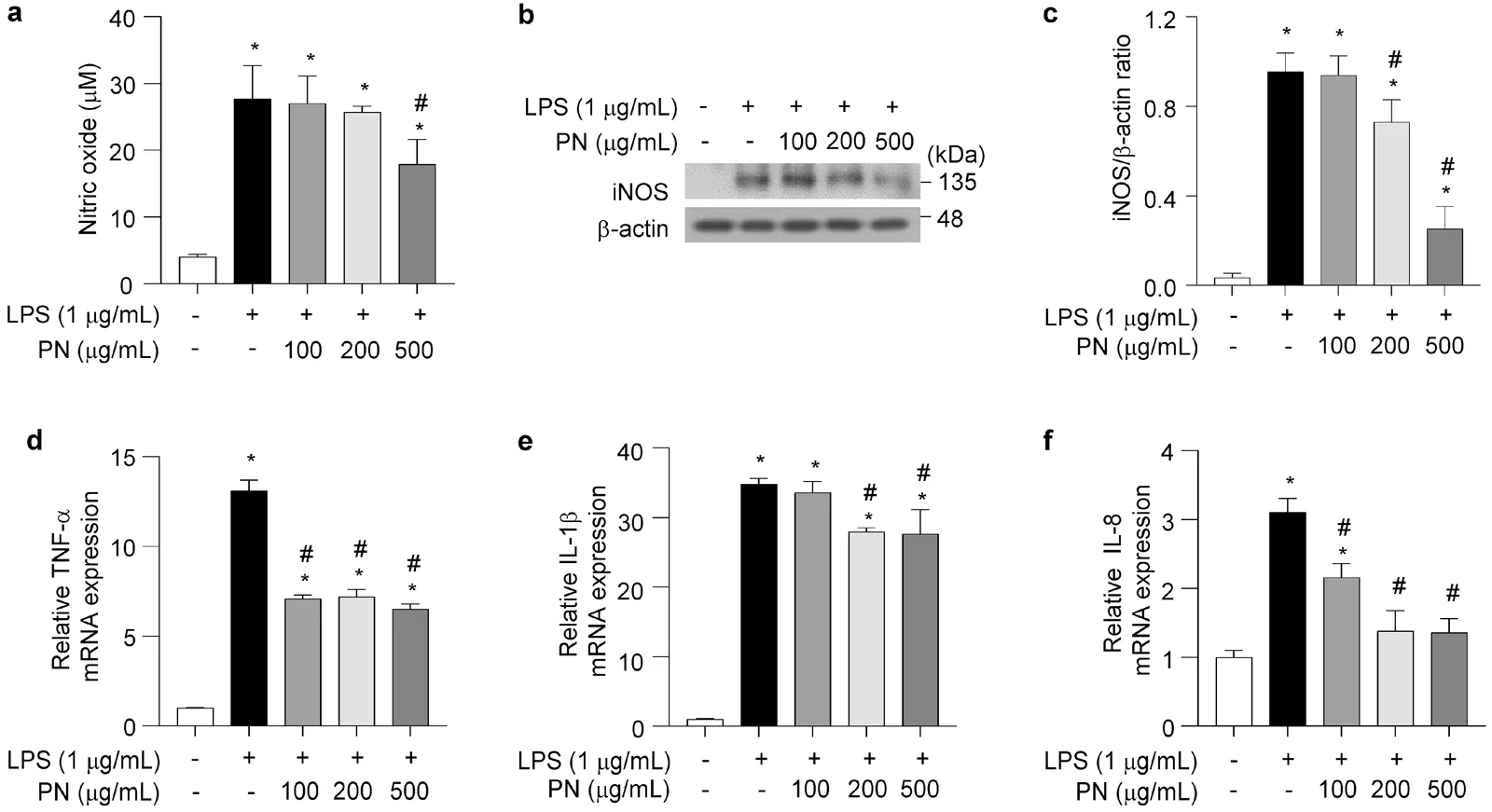
Effects of polynucleotides (PN) on lipopolysaccharide (LPS)-stimulated proinflammatory mediator production in RAW 264.7 cells. RAW 264.7 cells were pretreated with PN (0–500 μg/mL) for 1 h and then stimulated with LPS (1 μg/mL) for 24 h. (**a**) Nitric oxide (NO) levels in the culture supernatants were measured using the Griess reagent. (**b**) Protein expression of inducible nitric oxide synthase (iNOS) was analyzed by Western blotting. (**c**) Densitometric analysis of iNOS protein levels. (**d**–**f**) Messenger RNA (mRNA) levels of tumor necrosis factor-α (*TNF-α*), interleukin-1β (*IL-1β*), and interleukin-8 (*IL-8*) were measured by real-time reverse transcription polymerase chain reaction (RT-PCR). Data are shown as the mean ± standard deviation (SD) of three independent experiments. * *p* < 0.05 vs. the untreated group; # *p* < 0.05 vs. the LPS-only group.

To evaluate the anti-inflammatory activity of PN in macrophages, RAW 264.7 cells were stimulated with LPS (1 μg/mL) in the presence or absence of PN. LPS treatment significantly elevated nitric oxide (NO) production (27.7 ± 5.0 μM) compared to that in the untreated group (4.0 ± 0.4 μM, *p* < 0.05). However, PN pretreatment significantly suppressed NO levels in a dose-dependent manner, reducing the levels to 27.0 ± 4.1, 25.7 ± 0.9, and 17.9 ± 3.7 μM at concentrations of 100, 200, and 500 μg/mL, respectively (*p* < 0.05; Figure 2a). Western blot analysis revealed that LPS-induced iNOS protein expression was attenuated after PN treatment (Figure 2b,c). Additionally, PN treatment significantly decreased the mRNA expression levels of TNF-α, IL-1β, and IL-8. LPS treatment increased TNF-α expression (13.1 ± 0.6) compared to that of the untreated group (normalized to 1.0; *p* < 0.05), whereas treatment with 100, 200, and 500 μg/mL PN significantly reduced TNF-α to 7.1 ± 0.2, 7.2 ± 0.4, and 6.5 ± 0.3, respectively (*p* < 0.05; Figure 2d). Similarly, LPS treatment significantly increased IL-1β expression (34.8 ± 0.8) compared to that of the untreated group (normalized to 1.0; *p* < 0.05), whereas treatment with 100, 200, and 500 μg/mL PN reduced IL-1β to 33.6 ± 1.6 (*p* = 0.414), 27.9 ± 0.6 (*p* < 0.05), and 27.6 ± 3.5 (*p* < 0.05), respectively (Figure 2e). Furthermore, LPS treatment significantly increased IL-8 expression (3.1 ± 0.2) compared to that of the untreated group (normalized to 1.0; *p* < 0.05), whereas treatment with 100, 200, and 500 μg/mL PN significantly reduced IL-8 to 2.2 ± 0.2, 1.4 ± 0.3, and 1.4 ± 0.2, respectively (*p* < 0.05; Figure 2f).

### 3.3. Effects of PN on NF-κB Nuclear Translocation in LPS-Stimulated RAW 264.7 Cells

**Figure 3:**
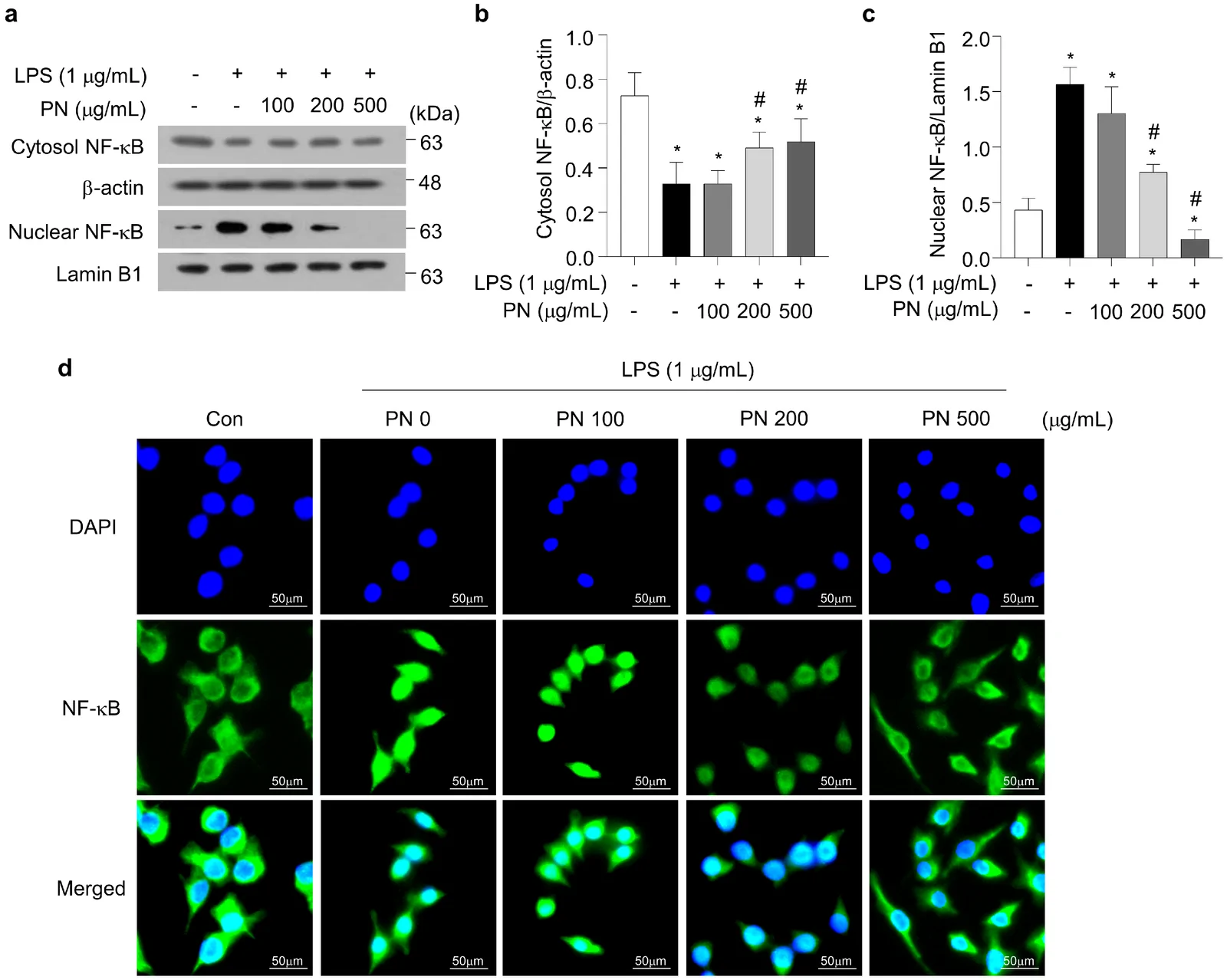
Effects of polynucleotides (PN) on nuclear factor-κB (NF-κB) nuclear translocation in lipopolysaccharide (LPS)-stimulated RAW 264.7 cells. Cells were pretreated with PN (0–500 μg/mL) for 1 h and then stimulated with 1 μg/mL LPS for 10 min. (**a**) NF-κB levels in cytosolic and nuclear fractions were assessed by Western blotting. β-actin and Lamin B1 were used as cytosolic and nuclear loading controls, respectively. (**b**,**c**) Quantification of cytosolic and nuclear NF-κB protein levels, normalized to β-actin and Lamin B1, respectively. (**d**) Representative immunofluorescence images showing NF-κB localization (green) and 4′,6-diamidino-2-phenylindole (DAPI)-stained nuclei (blue). Images were acquired at ×100 magnification. Data are presented as the mean ± standard deviation (SD) of three independent experiments. * *p* < 0.05 vs. the untreated control group; # *p* < 0.05 vs. the LPS-only group.

LPS stimulation induced nuclear translocation of NF-κB in RAW 264.7 cells. However, PN pretreatment significantly reduced the LPS-induced nuclear translocation of NF-κB and concomitantly increased cytosolic NF-κB in a dose-dependent manner (Figure 3a–c). Immunofluorescence staining confirmed that PN pretreatment attenuated the LPS-induced nuclear localization of NF-κB (Figure 3d), supporting an association between PN treatment and reduced LPS-triggered inflammatory signaling.

### 3.4. Inhibitory Effects of PN on TI-Stimulated Proinflammatory Cytokines in HaCaT Cells

**Figure 4:**
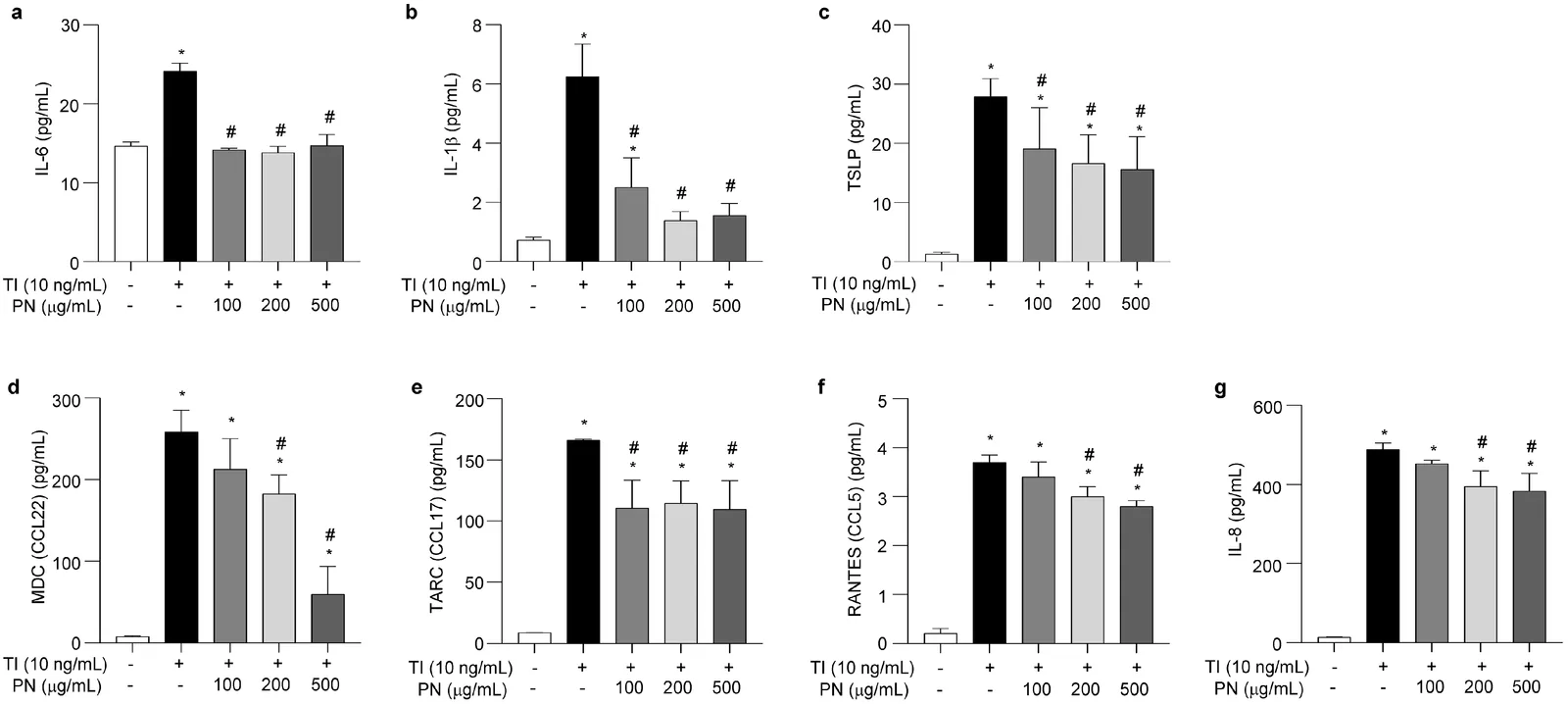
Effects of polynucleotides (PN) on tumor necrosis factor-α (TNF-α)/interferon-γ (IFN-γ) (TI)-induced cytokine and chemokine secretion in HaCaT cells. Cells were pretreated with PN for 1 h and then stimulated with 10 ng/mL TI for 24 h. (**a**–**g**) The concentrations of interleukin-6 (IL-6), interleukin-1β (IL-1β), thymic stromal lymphopoietin (TSLP), macrophage-derived chemokine (MDC; CCL22), thymus and activation-regulated chemokine (TARC; CCL17), regulated upon activation, normal T cell expressed and secreted (RANTES; CCL5), and interleukin-8 (IL-8) in culture supernatants were measured via enzyme-linked immunosorbent assay (ELISA). Data are presented as the mean ± standard deviation (SD) of three independent experiments. * *p* < 0.05 vs. the untreated control group; # *p* < 0.05 vs. the TI-only group.

We examined the effects of PN on the production of IL-6, IL-1β, and TSLP in TI-stimulated HaCaT cells. TI treatment significantly increased IL-6 levels (24.2 ± 1.0 pg/mL) compared to those of the untreated group (14.7 ± 0.5 pg/mL, *p* < 0.05), whereas treatment with 100, 200, and 500 μg/mL PN reduced IL-6 to 14.2 ± 0.2, 13.8 ± 0.8, and 14.7 ± 1.4 pg/mL, respectively (*p* < 0.05; Figure 4a). Similarly, TI treatment significantly increased IL-1β secretion levels (6.2 ± 1.1 pg/mL) compared to those of the untreated group (0.7 ± 0.1 pg/mL, *p* < 0.05). In contrast, treatment with 100, 200, and 500 μg/mL PN reduced IL-1β to 2.5 ± 1.0, 1.3 ± 0.3, and 1.6 ± 0.4 pg/mL, respectively (*p* < 0.05; Figure 4b). Furthermore, TI treatment increased TSLP secretion levels (27.9 ± 3.0 pg/mL) compared to those of the untreated group (1.3 ± 0.3 pg/mL, *p* < 0.05), whereas treatment with 100, 200, and 500 μg/mL PN significantly reduced TSLP to 19.1 ± 6.9, 16.6 ± 4.8, and 15.6 ± 5.5 pg/mL, respectively (*p* < 0.05; Figure 4c).

### 3.5. Inhibitory Effects of PN on TI-Stimulated Expression of Chemokines in HaCaT Cells

The effects of PN on the secretion of MDC (CCL22), TARC (CCL17), RANTES (CCL5), and IL-8 were evaluated in TI-stimulated HaCaT cells. TI treatment significantly increased MDC levels (258.8 ± 26.4 pg/mL) compared to those of the untreated group (7.6 ± 1.0 pg/mL, *p* < 0.05), whereas treatment with 100, 200, and 500 μg/mL PN reduced MDC to 212.9 ± 37.4 (*p* = 0.069), 182.6 ± 23.2 (*p* < 0.05), and 59.0 ± 34.1 pg/mL (*p* < 0.05), respectively (Figure 4d). Similarly, TI treatment significantly increased TARC levels (166.1 ± 0.8 pg/mL) compared to those of the untreated group (8.7 ± 0.1 pg/mL, *p* < 0.05), whereas treatment with 100, 200, and 500 μg/mL PN significantly reduced TARC to 110.6 ± 22.8, 114.6 ± 18.3, and 109.8 ± 23.2 pg/mL, respectively (*p* < 0.05; Figure 4e). Moreover, TI treatment significantly increased RANTES levels (3.7 ± 0.1 pg/mL) compared to those of the untreated group (0.2 ± 0.1 pg/mL, *p* < 0.05), whereas treatment with 100, 200, and 500 μg/mL PN reduced RANTES to 3.4 ± 0.3 (*p* = 0.076), 3.0 ± 0.2 (*p* < 0.05), and 2.8 ± 0.1 pg/mL (*p* < 0.05), respectively (Figure 4f). Finally, TI stimulation significantly increased IL-8 levels (488.4 ± 16.7 pg/mL) compared to those of the untreated group (13.0 ± 0.9 pg/mL, *p* < 0.05). In contrast, treatment with 100, 200, and 500 μg/mL PN reduced IL-8 to 452.6 ± 8.8 (*p* = 0.147), 394.4 ± 39.7 (*p* < 0.05), and 382.9 ± 44.4 pg/mL (*p* < 0.05), respectively (Figure 4g).

### 3.6. Effects of PN on the Phosphorylation of MAPK, JAK, and STAT in HaCaT Cells

**Figure 5:**
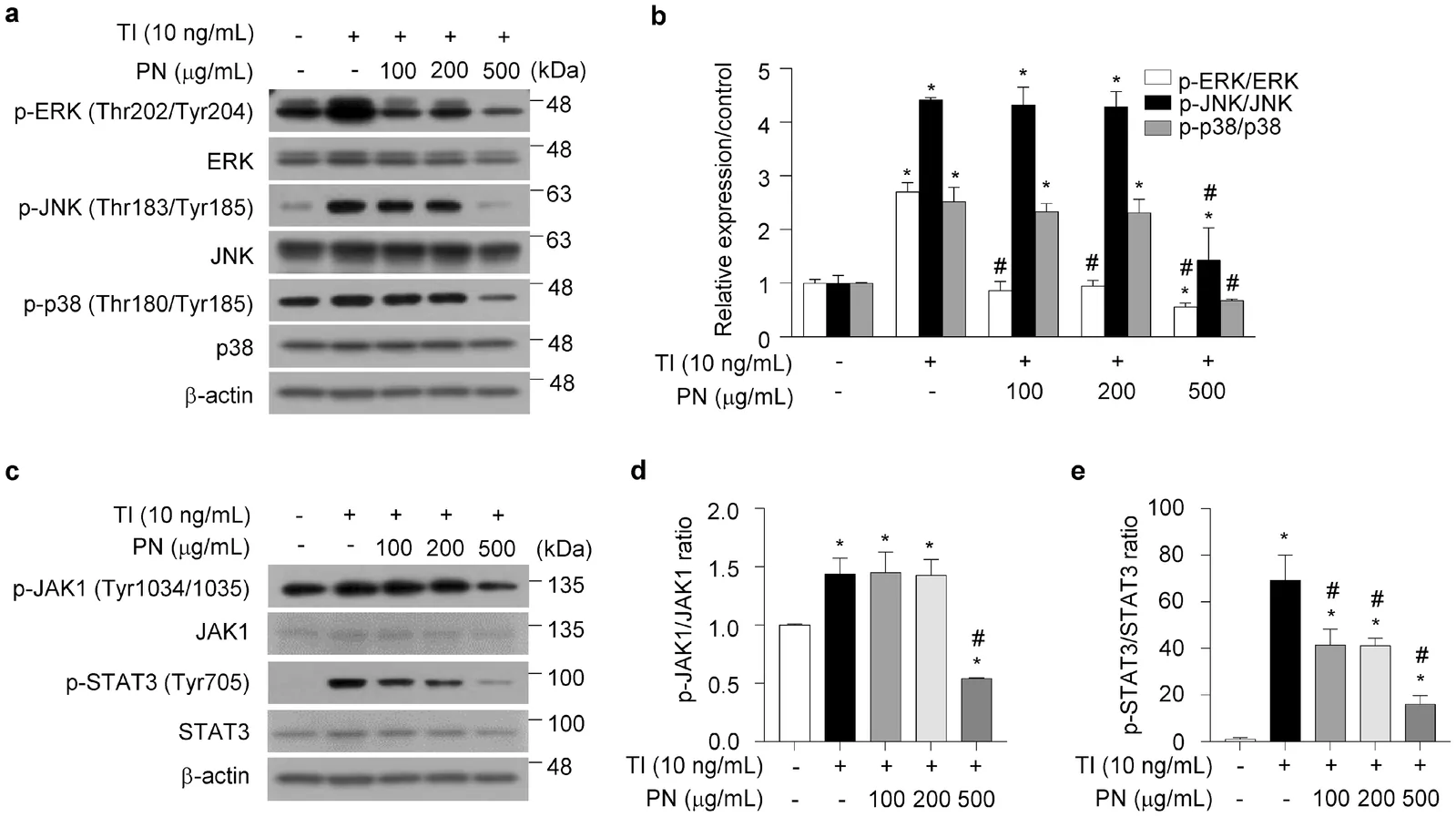
Effects of polynucleotides (PN) on tumor necrosis factor-α (TNF-α)/interferon-γ (IFN-γ) (TI)-stimulated phosphorylation of mitogen-activated protein kinase (MAPK) and Janus kinase/signal transducer and activator of transcription (JAK/STAT) signaling pathway proteins in HaCaT cells. Cells were pretreated with PN for 1 h and stimulated with 10 ng/mL TI for 30 min. (**a**) Protein levels of phosphorylated and total extracellular signal-regulated kinase (ERK), c-Jun N-terminal kinase (JNK), and p38 were assessed via Western blotting. (**b**) Densitometric quantification of phosphorylated ERK (p-ERK)/ERK, phosphorylated JNK (p-JNK)/JNK, and phosphorylated p38 (p-p38)/p38 protein expression ratios. (**c**) Protein levels of phosphorylated and total Janus kinase 1 (JAK1) and signal transducer and activator of transcription 3 (STAT3) were analyzed via Western blotting. (**d**,**e**) Quantification of phosphorylated JAK1 (p-JAK1)/JAK1 and phosphorylated STAT3 (p-STAT3)/STAT3 ratios. Data are presented as the mean ± standard deviation (SD) of three independent experiments. * *p* < 0.05 vs. the untreated control group; # *p* < 0.05 vs. the TI-only group.

TI stimulation increased the relative phosphorylation levels of ERK (2.7 ± 0.2), JNK (4.4 ± 0.1), and p38 (2.5 ± 0.3) compared to those of the untreated control group (normalized to 1.0; *p* < 0.05; Figure 5a,b). Notably, treatment with 500 μg/mL PN significantly reduced the phosphorylation of these kinases, yielding levels of 0.6 ± 0.1 for ERK, 1.4 ± 0.6 for JNK, and 0.7 ± 0.1 for p38 (*p* < 0.05). Similarly, the relative phosphorylation levels of JAK1 (1.4 ± 0.1) and STAT3 (69.1 ± 10.8) were elevated by TI stimulation compared to those of the untreated control group (normalized to 1.0; *p* < 0.05; Figure 5c–e). However, treatment with 500 μg/mL PN significantly reduced JAK1 and STAT3 phosphorylation to 0.5 ± 0.1 and 16.1 ± 3.6, respectively (*p* < 0.05).

### 3.7. Effects of PN on TI-Stimulated NF-κB Activation in HaCaT Cells

**Figure 6:**
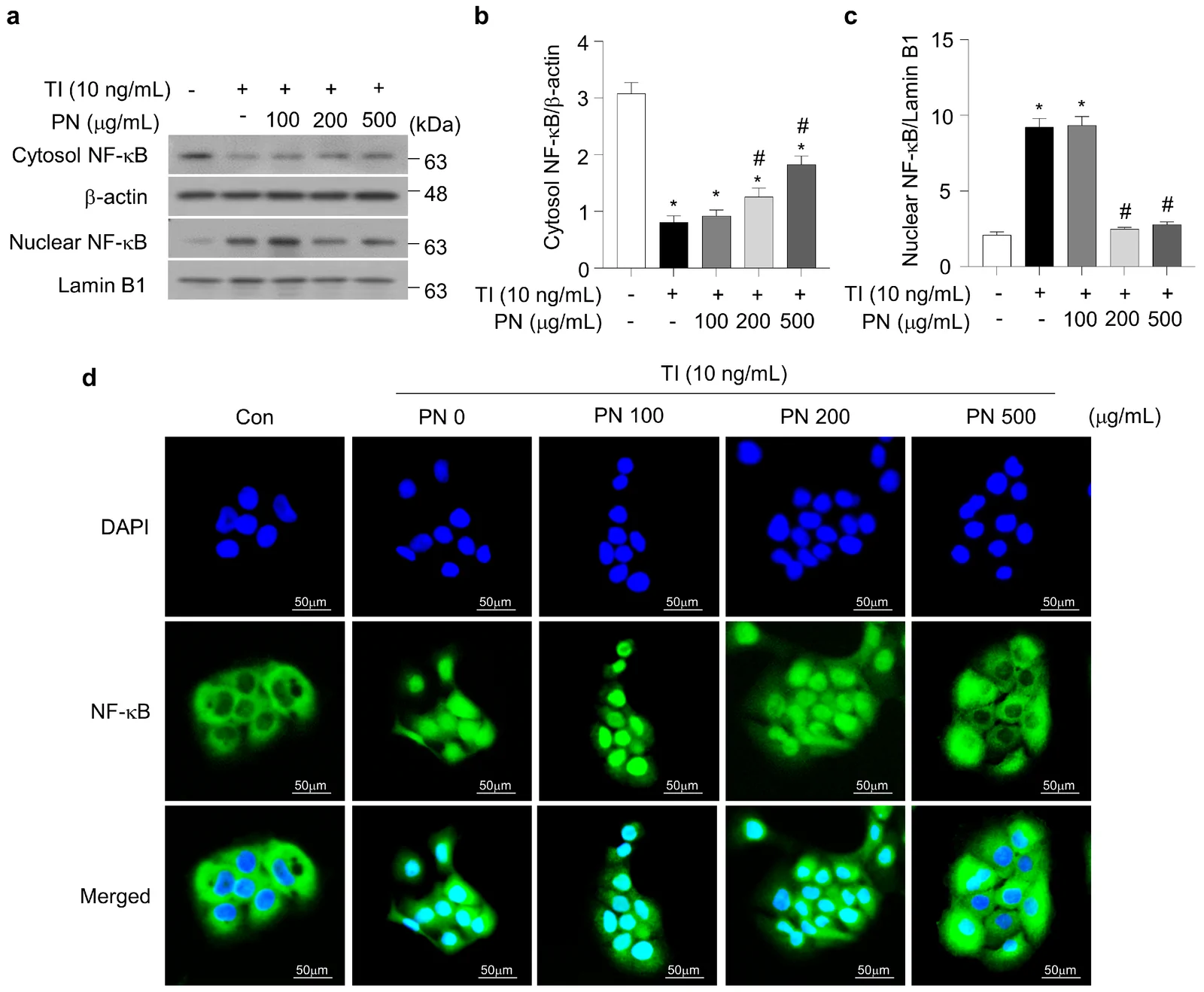
Effects of polynucleotides (PN) on nuclear factor-κB (NF-κB) nuclear translocation in tumor necrosis factor-α (TNF-α)/interferon-γ (IFN-γ) (TI)-stimulated HaCaT cells. Cells were pretreated with PN for 1 h (100–500 μg/mL) and then stimulated with 10 ng/mL TI for 10 min. (**a**) Western blot analysis of NF-κB levels in cytosolic and nuclear fractions. β-actin and Lamin B1 were used as cytosolic and nuclear loading controls, respectively. (**b**,**c**) Quantification of cytosolic and nuclear NF-κB protein levels, normalized to β-actin and Lamin B1, respectively. (**d**) Representative immunofluorescence images showing NF-κB localization (green) and 4′,6-diamidino-2-phenylindole DAPI-stained nuclei (blue). Images were acquired at ×100 magnification. Data are presented as the mean ± standard deviation (SD) of three independent experiments. * *p* < 0.05 vs. the untreated control group; # *p* < 0.05 vs. the TI-only group.

Stimulation with 10 ng/mL TI significantly decreased cytosolic NF-κB and increased nuclear NF-κB levels compared to those of the untreated group (Figure 6a). However, pretreatment with PN led to a dose-dependent retention of cytosolic NF-κB and a corresponding reduction in nuclear NF-κB levels. Quantitative analysis showed that the cytosolic NF-κB/β-actin ratio decreased to 0.8 ± 0.1 upon TI stimulation, whereas PN treatment restored this level to 1.3 ± 0.2 at 200 μg/mL and 1.8 ± 0.1 at 500 μg/mL (*p* < 0.05; Figure 6b). Conversely, the nuclear NF-κB/Lamin B1 ratio increased to 9.2 ± 0.6 following TI stimulation and was significantly reduced to 2.5 ± 0.1 at 200 μg/mL and 2.8 ± 0.2 with 500 μg/mL PN treatment (*p* < 0.05; Figure 6c). Immunofluorescence microscopy corroborated the reduced nuclear NF-κB translocation after PN treatment (Figure 6d).

## 4. Discussion

Atopic dermatitis (AD) is a multifactorial chronic inflammatory skin disease characterized by epidermal barrier dysfunction, immune imbalance, and complex interactions among genetic and environmental triggers [22,23]. A primary initiating event is epidermal barrier impairment, which elevates transepidermal water loss and facilitates the penetration of irritants and allergens [24]. This breach triggers the innate immune system and drives a skewed T helper 2 (Th2)-type adaptive immune response. Beyond conventional cytokine signaling, macrophage-derived extracellular vesicles can amplify type 2 immunity by potentiating group 2 innate lymphoid cell (ILC2) function in allergic inflammation [25], suggesting that vesicle-mediated intercellular communication may similarly operate in AD. Furthermore, keratinocytes actively contribute to this inflammatory milieu by secreting cytokines and chemokines that recruit and activate infiltrating immune cells, thereby escalating cutaneous inflammation [26,27]. The causal primacy of immune dysregulation in cutaneous inflammation is illustrated by immune-related adverse events: blockade of programmed cell death protein 1 (PD-1)/programmed death-ligand 1 (PD-L1) checkpoints alone can precipitate inflammatory skin and muscle disease [28]. This indicates that loss of inhibitory immune signaling is sufficient to initiate cutaneous inflammatory pathology, reinforcing the rationale for strategies that restore inhibitory or pro-resolving signals rather than merely neutralizing individual effector cytokines.

In the present study, we demonstrate that polynucleotides (PN)—highly purified DNA polymers derived from salmonid gonads—exhibit pronounced anti-inflammatory effects in two established in vitro models: TNF-α/IFN-γ (TI)-stimulated HaCaT keratinocytes and LPS-stimulated RAW 264.7 macrophages. These dual cell models recapitulate key features of AD pathophysiology, including epithelial cytokine responses and macrophage-driven inflammatory signaling [3,29].

The primary contribution of the present study is the cell-type-resolved characterization of PN-associated anti-inflammatory responses. Our previous in vivo study demonstrated marked improvement in AD-like skin lesions after topical PN treatment; however, whole-tissue analyses could not distinguish direct effects on individual cell populations from indirect effects resulting from improved hydration, barrier restoration, or microenvironmental shifts. By evaluating keratinocytes and macrophages independently under defined stimulation conditions, the present study provides evidence that PN directly modulate distinct yet complementary cellular responses: macrophage-associated NO production, iNOS expression, and proinflammatory gene transcription, as well as keratinocyte-derived cytokines and chemokines involved in epithelial inflammation and immune-cell recruitment. Concomitant changes in NF-κB nuclear localization and MAPK/JAK1/STAT3 phosphorylation provide molecular correlates that may partly explain the previous in vivo observations. Crucially, these findings should be interpreted as a cell-type-resolved molecular extension and hypothesis-generating evidence rather than definitive identification of a receptor-specific or causal mechanism.

In RAW 264.7 macrophages, PN significantly suppressed LPS-induced nitric oxide (NO) accumulation and iNOS protein expression. Furthermore, PN attenuated the mRNA expression of TNF-α, IL-1β, and IL-8. These mediators are pivotal in acute inflammatory responses and serve as upstream regulators of broader cytokine cascades [30]. However, since TNF-α, IL-1β, and IL-8 were assessed only at the mRNA level, the present findings do not establish corresponding reductions in their protein production or secretion. Nevertheless, the reductions in NO production and iNOS protein expression provide complementary evidence of the anti-inflammatory activity of PN. The concurrent inhibition of NO and iNOS suggests that PN may regulate redox-sensitive signaling during macrophage activation. PN pretreatment was associated with attenuated LPS-induced nuclear translocation of NF-κB; however, this observation does not establish direct inhibition of the NF-κB pathway.

In TI-stimulated HaCaT keratinocytes, PN significantly reduced the secretion of IL-6, IL-1β, TSLP, and chemokines including MDC/CCL22, TARC/CCL17, RANTES/CCL5, and IL-8—all of which are implicated in T cell recruitment and AD pathogenesis [7,12,31,32]. PN treatment was associated with reduced activation of multiple proinflammatory signaling pathways. Specifically, PN treatment reduced the phosphorylation of MAPKs (ERK, JNK, and p38), which are central to the regulation of proinflammatory gene expression in both immune and epithelial cells. PN treatment was also associated with reduced JAK1 and STAT3 phosphorylation; however, these changes do not definitively provide evidence of direct inhibition of the JAK1/STAT3 pathway. Since the JAK/STAT axis is therapeutically relevant to AD, these findings warrant further investigation of whether PN-associated signaling changes contribute causally to the observed anti-inflammatory effects.

Although the primary molecular target of PN was not examined in the present study, the adenosine A2A receptor (A2AR) represents a plausible candidate. Polydeoxyribonucleotide (PDRN), a related salmonid-derived DNA polymer, exerts A2AR-dependent anti-inflammatory effects in experimental colitis and psoriasis; in the psoriasis model, these effects were associated with suppression of NF-κB signaling [19,33]. These observations suggest that A2AR-associated signaling may contribute to the anti-inflammatory activity observed in our models. However, since PN and PDRN differ in molecular weight distribution and receptor-specific experiments were not performed, A2AR should be regarded as a candidate rather than a confirmed direct target. Further studies using A2AR antagonism, genetic silencing, and ligand-binding assays are required to establish receptor-level causality.

Another distinct advantage of PN is their favorable biocompatibility and well-documented regenerative potential. Prior studies have shown that PN promote cellular proliferation and wound healing via extracellular matrix remodeling and hydration [34]. Reparative macrophages contribute to cutaneous repair through pro-angiogenic mediators acting on the hypoxia-inducible factor 1-α and vascular endothelial growth factor A (HIF-1α/VEGFA) axis [35], raising the possibility that an agent that dampens classical macrophage activation while preserving reparative programs could couple anti-inflammatory and reparative outcomes. This dual functionality—combining anti-inflammatory and regenerative effects—may offer additional therapeutic benefits in AD, where restoring barrier integrity is as crucial as controlling inflammation [21]. Furthermore, the non-cytotoxic nature of PN at therapeutically effective doses, as confirmed in our study, supports their translational potential.

Despite the promising in vitro anti-inflammatory effects observed, this study has several limitations that warrant acknowledgment. First, the exclusive use of immortalized cell lines (HaCaT and RAW 264.7) does not fully reproduce human cell physiology, immune–epithelial interactions, or the dynamic skin microenvironment. Second, the present models reproduce selected AD-relevant inflammatory responses; however, they do not recapitulate the full immunological and epidermal barrier phenotype of AD. Specifically, IL-4/IL-13-driven type 2 inflammatory conditions were not modeled, nor were key epidermal differentiation and barrier-related proteins—such as filaggrin, involucrin, and claudins—assessed. Therefore, whether PN directly modulates type 2 inflammation or restores epidermal barrier function remains to be determined. Although our previous work demonstrated in vivo efficacy in a DNCB-induced mouse model [21], future studies should incorporate IL-4/IL-13 stimulation, primary human keratinocytes, primary human macrophages, and other relevant immune cells; human three-dimensional skin equivalents; patient-derived organoids; and assessments of epidermal differentiation and tight-junction proteins. Experimental validation using lesional or non-lesional skin biopsies from patients with AD will also be required to establish clinical relevance. Third, the direct molecular targets of PN remain unknown, and the observed reductions in NF-κB nuclear localization and MAPK/JAK1/STAT3 phosphorylation do not establish direct pathway inhibition or causal involvement. Since pathway-specific inhibition, knockdown, and rescue experiments were not performed, these observations demonstrate associations with attenuated signaling activation rather than direct pathway inhibition. Future studies using these approaches, together with direct target validation, will be required to establish whether the observed signaling changes mediate the anti-inflammatory effects of PN. Whether PN act through intracellular nucleic acid-sensing pathways also warrants further investigation. Fourth, the present study relied on targeted assessment of a predefined panel of cytokines, chemokines, and signaling proteins, rather than unbiased transcriptomic or proteomic profiling. Consequently, potential off-target effects, broader pathway involvement, or additional mediators of PN activity beyond the molecules examined here cannot be ruled out; future studies incorporating high-throughput omics profiling approaches will be needed to comprehensively define the mechanism of action of PN. Fifth, the absence of a pharmacological positive control prevents direct comparison of PN efficacy with that of approved AD treatments. Future studies should include head-to-head comparative assays with established anti-inflammatory agents. Sixth, the present study evaluated only acute cellular responses after a single PN pretreatment. Therefore, the durability of the anti-inflammatory effects under repeated or prolonged exposure, as well as potential rebound effects after treatment withdrawal, remains to be determined. Future studies incorporating extended time-course analyses, repeated or intermittent dosing, washout and re-challenge experiments, and post-treatment relapse monitoring will be required to establish the durability and long-term therapeutic relevance of PN treatment.

## 5. Conclusions

PN treatment significantly attenuates the production of proinflammatory mediators in stimulated HaCaT keratinocytes and RAW 264.7 macrophages. This effect is associated with reduced NF-κB nuclear localization and MAPK/JAK1/STAT3 phosphorylation. Nevertheless, these in vitro findings should be interpreted with caution, given the exclusive use of immortalized cell lines, the lack of receptor-specific mechanistic data, the absence of transcriptomic or proteomic confirmation, and the lack of validation in primary human AD tissue in the present study. Although these cellular data support further preclinical investigation, they do not establish therapeutic efficacy in AD. Validation in translationally relevant in vivo disease models, followed by studies using human skin tissues and clinical studies, is required to determine the translational relevance, safety, and optimal delivery of PN in AD management.

## Data Availability

The raw data supporting the findings and conclusions of this study are available from the corresponding author upon reasonable request.

## Supplementary Materials

The following supporting information can be downloaded at: https://www.mdpi.com/article/10.3390/biomedicines14081826/s1, Supplementary Table S1. Primer sequences used for real-time RT-PCR.
