# Supplementary material for: Polynucleotides Attenuate Atopic Dermatitis-like Inflammatory Signaling in Keratinocytes and Macrophages
Source: Biomedicines. 2026 Aug 13;14(8):1826. doi: 10.3390/biomedicines14081826 (PMC13509240; doi:10.3390/biomedicines14081826)
Supplement: Supplementary file 1 [file biomedicines-14-01826-s001.zip › biomedicines-4439378-supplementary.pdf]

Table S1. Primer sequences used for real time reverse transcription polymerase chain reaction.

Genes	Sequence (5'→3')	Product size (bp)
Mouse IL-1β	Forward: TGGACCTTCCAGGATGAGGACA<br>Reverse: GTTCATCTCGGAGCCTGTAGTG	148
Mouse IL-8	Forward: GGTGATATTCGAGACCATTTACTG<br>Reverse: GCCAACAGTAGCCTTCACCCAT	112
Mouse TNF-α	Forward: GGTGCCTATGTCTCAGCCTCTT<br>Reverse: GCCATAGAACTGATGAGAGGGAG	139
Mouse GAPDH	Forward: CATCACTGCCACCCAGAAGACTG<br>Reverse: ATGCCAGTGAGCTTCCCGTTCAG	153

IL, Interleukin; TNF-α, Tumor Necrosis Factor-Alpha; GAPDH, Glyceraldehyde-3-Phosphate Dehydrogenase.
